# Vitamin D sensitizes cervical cancer to radiation-induced apoptosis by inhibiting autophagy through degradation of Ambra1

**DOI:** 10.1038/s41420-024-02279-7

**Published:** 2025-01-04

**Authors:** Zhaoming zhang, Xinyue Yu, Guanghui Cheng

**Affiliations:** Department of Radiation Oncology, China-Japan Union Hospital of Jilin University, Changchun, China

**Keywords:** Radiotherapy, Cancer therapeutic resistance

## Abstract

Cervical cancer (CC) is becoming a major health issue globally, and radiotherapy plays a crucial role in its treatment. However, the prognosis of some patients remains poor due to tumor resistance to the therapy. This study aimed to explore whether vitamin D could confer a more radiosensitive phenotype in CC based on our previous findings and detection using the database. We found that vitamin D sensitized vitamin D receptor (VDR)-positive CC cells (Siha and Caski) to the cytotoxic effects of radiation in vivo and in vitro. We examined conventional radiation-induced cell death, such as DNA damage and cell cycle arrest, in vitamin D–treated cells to detect the underlying mechanism, but no association was observed between them. Subsequently, our proteome analysis exhibited that autophagy was reduced in irradiated CCs treated with vitamin D, and apoptosis displayed the opposite effect. Moreover, we confirmed that vitamin D-pretreated irradiated cells displayed reduced autophagy activity mediated by the Ambra1 downregulation, and the elevation of apoptosis was attributed to the activation of caspase 8. Importantly, the pharmacological inhibition of caspases or the Ambra1 overexpression could restore tumor proliferation under the vitamin D and radiation combination treatment. Hence, the aforementioned findings revealed the essential impact of vitamin D in terms of enhancing radiosensitivity in CC meditated by inhibiting autophagy and proposed the addition of vitamin D as a viable strategy to improve the therapeutic efficacy of VDR-positive CC.

## Introduction

Cervical cancer (CC) is the fourth most common female malignant tumor globally [1], and radiotherapy plays an essential role in treating all stages of CC. However, a poor therapeutic effect still exists owning to radiation resistance, and the cause of this resistance remains to be unveiled. Therefore, exploring the molecular mechanism of radioresistance to CC and looking for radiosensitizers are worthwhile in improving CC therapy efficacy. Various preclinical trials of radiosensitizers intended to find the combination therapy which would be less toxic than radiation alone while maintaining therapeutic preference [2], but expected less toxicity has not been ascertained. This emphasizes the unmet need for credible adjuvants.

Frankly speaking, the mechanism of tumor cell death is complicated. As exogenous stress, radiotherapy can initiate multiple signal pathways simultaneously, so the phenomenon of cell death observed in cancer cells may be a “mixed” form of cell death, which does not simply correspond to a particular type of cell death. The traditional modes of cell death caused by radiation include DNA double-strand damage and G2/M phase arrest. Autophagy can also occur under stress as an evolutionarily conservative process, such as in irradiated tumor cells. Autophagy was initially found in yeast as an ability to maintain cell survival under pressure by circulating intracellular components. However, in higher eukaryotes, the mechanism of autophagy regulating cell survival or death is much more complex, especially in cancer cells [3]. It is supposed that the irradiated tumor cells might alleviate the cytotoxicity caused by external pressure via autophagy to maintain intracellular dynamic balance. If autophagy cannot restore the dynamic balance, tumor cells can react by activating the apoptosis process. Of note, the protein networks of autophagy and apoptosis are highly correlated [4]. Therefore, the mutual regulation between autophagy and apoptosis implies that tumors can make a more adaptive response to stress signals.

Studies of autophagy modulation have shown that additive vitamin D in tumor treatment resulted in enhanced therapeutic efficacy [5, 6], indicating that autophagy regulation mediated by vitamin D might be a potential adjuvant regimen. Moreover, our previous study has demonstrated that vitamin D-adjuvant radiation produced an amplified treatment effect in colorectal cancer [7]. Vitamin D can bind to vitamin D receptor (VDR) and form the VDR-RXR complex to translocate to the nuclear thus ensuring proliferation inhibition required for vitamin D [8]. In addition, vitamin D can also function in the non-genome signaling pathway mediated by 1, 25D-MARRS, an acute response protein [9]. Up till now, the role of vitamin D in CC therapy has not been definitely explored.

In this study, we analyzed the level of VDR in patients and found a close relationship between VDR and the prognosis of CC. Accordingly, we demonstrated that vitamin D sensitized the CC to radiation in vitro and in vivo. In this context, our proteome results revealed that autophagy and apoptosis exhibited a significant role in the inhibition of tumors through their ability to modulate pro-survival or pro-death genes. Independent of single cell death, the decreased expression of Ambra1 and the increased expression of caspases highlighted a more complex relationship between apoptosis and autophagy. Ultimately, our study reflected that vitamin D-induced modulation of autophagy might be harnessed for the radiotherapeutic benefit of CC.

## Results

### VDR correlated with cervical cancer

**Fig. 1:**
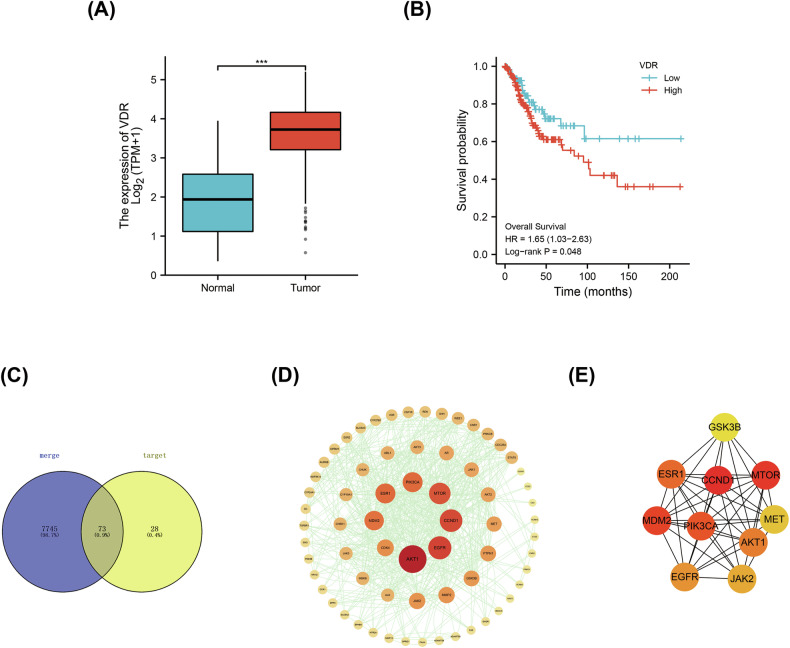
VDR correlated with cervical cancer. **A** The expression of VDR in the TCGA database of 306 CC tumors and 3 adjacent tissues. **B** Survival analysis of patients detected in TCGA database. **C** Venn diagram of common targets between vitamin D and CC. **D** The PPI network of common targets was constructed using Cytoscape. **E** The PPI network of core targets generated from (**D**) by using cytohubba of Cytoscape.

Vitamin D level has been reported to be associated with cancer risk, and it plays distinct roles in various tumors [10]. Based on our previous study, we determined to see what function it performed in CC. As the classic VDR, VDR was first searched in the TCGA database. It was found that the expression level of VDR in CC was significantly higher than that in adjacent tissues (Fig. 1A), and the same results were found in the GEPIA and Oncomine database (Fig. S1A, B), which indicated that tumor cells would have a superior response to vitamin D than the normal tissues. In addition, patients with higher expression of VDR had a poorer prognosis than those with VDR-low in CC (Fig. 1B), suggesting that VDR appeared to be a potential target and those refractory patients with relatively high VDR expression might respond more actively to vitamin D treatment. Furthermore, we discovered many common targets between vitamin D and CC through data network integration, and the core target sites might be associated with AKT, mTOR, and EGFR (Fig. 1C–E). These data suggested a close correlation between vitamin D and CC. Next, we detected the expression of VDR in CC cell lines Siha and Caski. (Fig. S1C). The above results indicated that vitamin D was closely related to the risk of CC and had the potential to be used as an adjuvant to enhance the therapeutic effect of CC.

### Vitamin D enhanced the radiosensitivity of cervical cancer

**Fig. 2:**
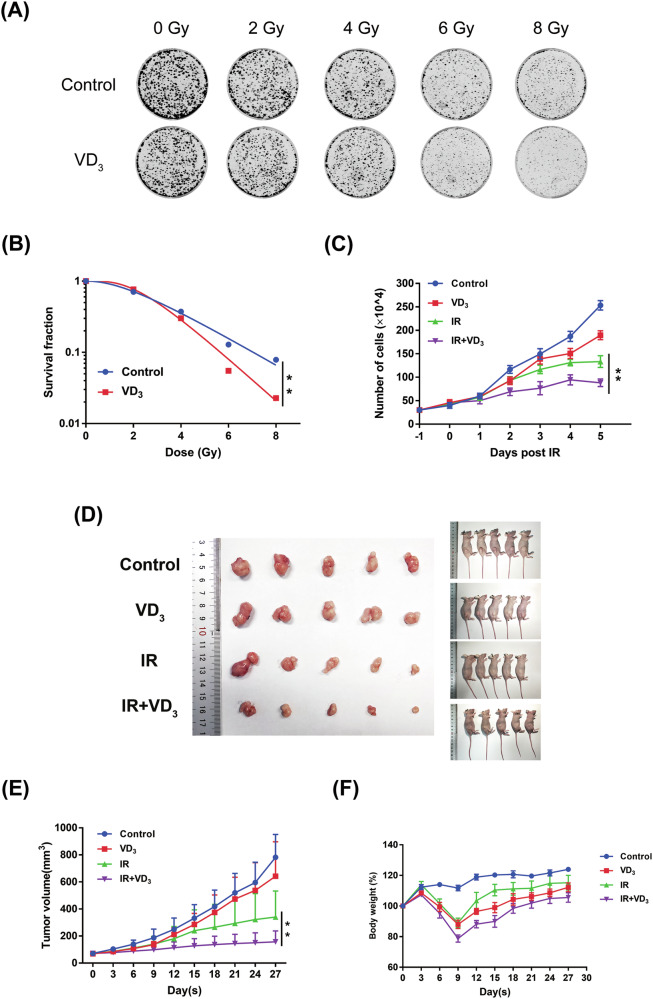
Vitamin D enhanced the radiosensitivity of cervical cancer. **A** The results of clone formation. A certain number of Siha cells were seeded in 6-well plates and then treated with radiation 0, 2, 4, 6, and 8 Gy or combined with vitamin D; 2 weeks later, crystal violet staining was utilized to calculate the number of clones. The survival fraction was calculated by clicking the multi-target model to fit the cell survival curve. **B** The survival score curve of the obtained clone number was forged according to the single-target multi-hit model *y* = 1 − (1−exp (−k*x))^N. **C** Siha cells were planted in a 6-well plate and dealt with different conditions. Within 5 days post-treatment, the number of surviving cells was measured by trypan blue staining every day. **D** Siha cells were injected into the right thigh of mice and were randomly divided into four groups: control, vitamin D, radiotherapy, and combined group, and dealt with the intended treatments. **E** Tumor picture and gross picture of mice. **F** Bodyweight of the mice. *p < 0.05, **p < 0.01, ***p < 0.001, ****p < 0.0001.

To confirm the radiosensitizing effect of vitamin D in CC, we firstly carried out the colony formation and found that the size and number of clones in the group without vitamin D treatment were significantly larger than that treated with vitamin D under different radiation doses (Fig. 2A). Furthermore, we calculated that the survival fraction of the vitamin D-untreated group was considerably higher than that of the treated group (Fig. 2B). Then trypan blue staining was used to count the number of surviving cells treated with vitamin D combined with 6 Gy for 1–5 days. The results showed that vitamin D alone exerted no significant suppression on the proliferation of Siha; however, the tumor inhibitory effect was significantly magnified in a time-dependent manner when combined with radiation (Fig. 2C). A similar radiosensitization tendency was confirmed in Caski cells (Fig. S2).

It has been proved that vitamin D can improve the radiosensitivity of CC in vitro. We intended to verify this radiosensitization in vivo. Consistent with the in vitro results, the inhibitory effect of either treatment alone on tumors was not noticeable; however, the remarkable tumor inhibition occurred when dealt with combination treatment (Fig. 2D, E). Additionally, the combination of vitamin D and radiation did not show a significant influence on the bodyweight of mice (Fig. 2F). The main organs of mice were detected by HE to evaluate the tissue toxicity, and no noticeable morphological changes were found among different groups (Figure S3). The above results demonstrated that vitamin D combined with radiation could play an effective impact on tumor inhibition in CC without additional toxic effects.

### Combination treatment did not affect DNA damage and G2/M arrest

**Fig. 3:**
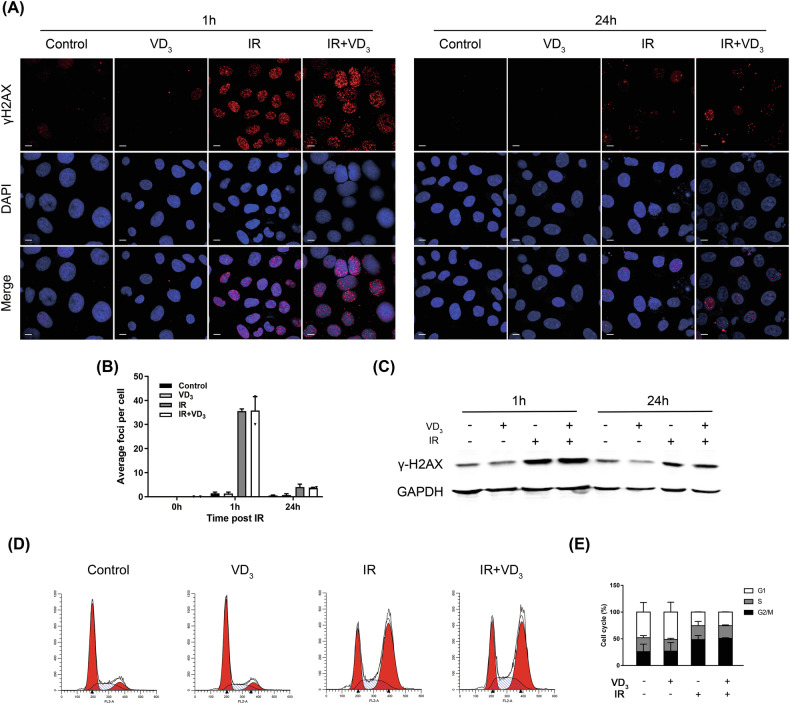
Combination treatment did not affect DNA damage and G2/M arrest. **A** Siha cells were seeded in confocal dishes and were ministered with control, vitamin D, radiation, and combined treatment after the cells were attached to the wall. After 1 h and 24 h, the γH2AX foci were determined by immunofluorescence. Scale bar: 20 μm. **B** Quantitative analysis of γH2AX foci. Three visual fields were selected to count the number in each group. **C** γH2AX expression was detected by western blot. **D** Cell cycle analysis. Cells treated with the indicated treatments were stained with PI and then analyzed by flow cytometry. **E** Quantitative analysis of the percentages of all phases. *p < 0.05, **p < 0.01, ***p < 0.001, ****p < 0.0001.

DNA damage plays a vital role in radiation-induced tumor cell demise, and the repairability of DNA damage determines the therapeutic effect of radiation [11]. We detected the immunofluorescence of γH2AX, a marker of DNA damage. The results showed that compared with the unirradiated groups, the formation of intracellular γH2AX foci in the irradiated groups increased significantly within 1 h post-irradiation; however, there was no significant difference in the attendance or absence of vitamin D (Fig. 3A, B), and the γH2AX foci in all groups gradually faded after 24 h. The level of γH2AX detected by western blot showed a similar trend with the immunofluorescence results (Fig. 3C). These results suggested that radiation could induce γH2AX in tumor cells, but vitamin D did not strengthen the radiation-triggered DNA damage in CC.

It is noticeable that the G2/M phase of tumor cells is the most sensitive phase to radiotherapy [12]. Therefore, previous studies are commonly focused on how to increase the G2/M phase ratio to improve the treatment response. In order to determine whether vitamin D could lead to G2/M phase arrest, we examined the cell cycle distribution with different treatments. The results revealed that independent of radiation, the G2/M phase ratio of the vitamin D-treated group was similar to the vitamin D-untreated groups (Fig. 3D, E), indicating that the radiosensitization effect induced by vitamin D was not attributed to the distribution of G2/M phase. Otherwise, there was a particularly much higher percentage of G2/M phase in response to radiation, which was consistent with the prior studies.

### The proteomic sequencing suggested changes in apoptosis and autophagy

**Fig. 4:**
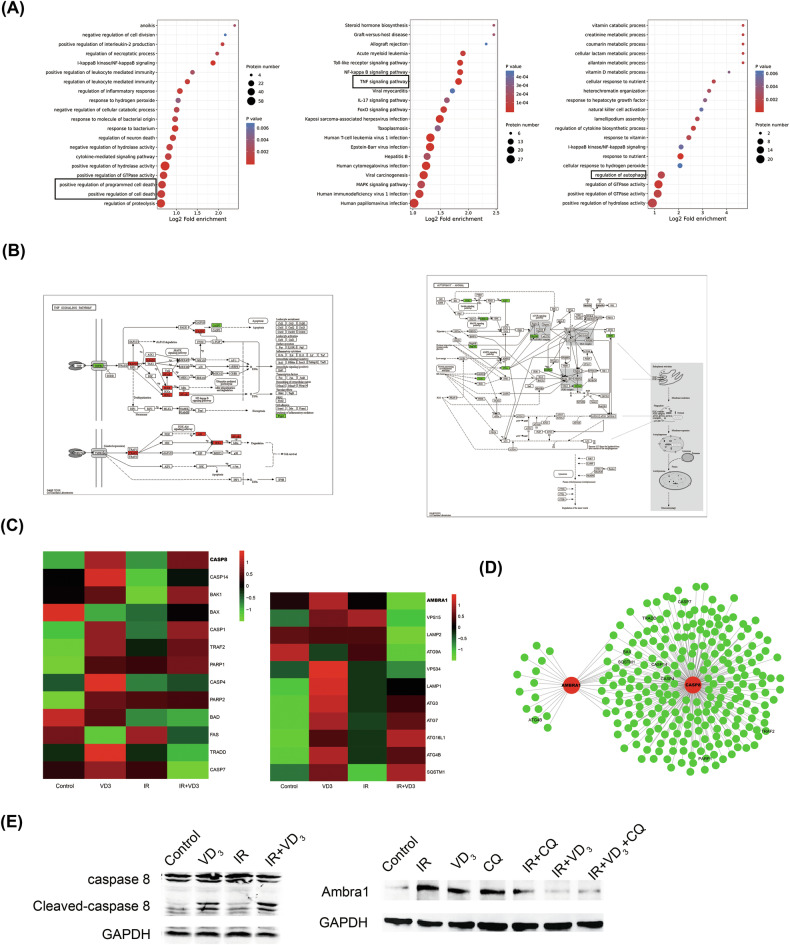
The proteomic sequencing suggested changes in apoptosis and autophagy. **A** Functional enrichment analysis of differentially expressed proteins by GO. The circle size represents the number of enriched proteins in each classification. Each color represents a different *p*-value. **B** KEGG signal pathway analysis. **C** The heat map of differentially expressed proteins under different treatments. **D** The protein interaction network between caspase 8 and Ambra1. **E** Western blot validation of caspase 8 and Ambra1.

In order to further elucidate the molecular mechanism of vitamin D in enhancing the radiosensitivity of CC, proteomic sequencing was used to detect the differentially expressed protein of Siha cells under different treatments. GO classification and enrichment analysis results displayed that programmed cell death, TNF signal pathway, and autophagy regulation were significantly enriched (Fig. 4A). Consistent with GO enrichment, KEGG analysis revealed that many of the proteins related to the apoptosis signaling pathway were significantly up-regulated, conversely, those proteins related to the autophagy pathway were remarkably down-regulated (Fig. 4B). Furthermore, we collected the differentially expressed proteins related to apoptosis and autophagy in the proteome data and constructed a heatmap to observe the protein alteration (Fig. 4C). It was found that there were abundant interaction pathways between apoptosis-related protein caspase 8 and autophagy-related protein Ambra1 by the interaction network analysis (Fig. 4D). Furthermore, we detected the expression of these two proteins by western blot and found that the results were consistent with the proteomics data (Fig. 4E). To further display the alteration of autophagy flux, we utilized autophagy inhibitor CQ to block autophagy degradation and found that the downregulation of Ambra1 was more obvious in the combination group (Fig. 4E). These results demonstrated that the mechanism of vitamin D-induced radiosensitization in CC was closely associated with apoptosis and autophagy, and there was a specific correlation between them which might be mediated by the reciprocal regulation between caspase 8 and Ambra1. However, the precise mechanism still needs to be further investigated.

### Vitamin D combining with radiation increased apoptosis and inhibited autophagy in cervical cancer

**Fig. 5:**
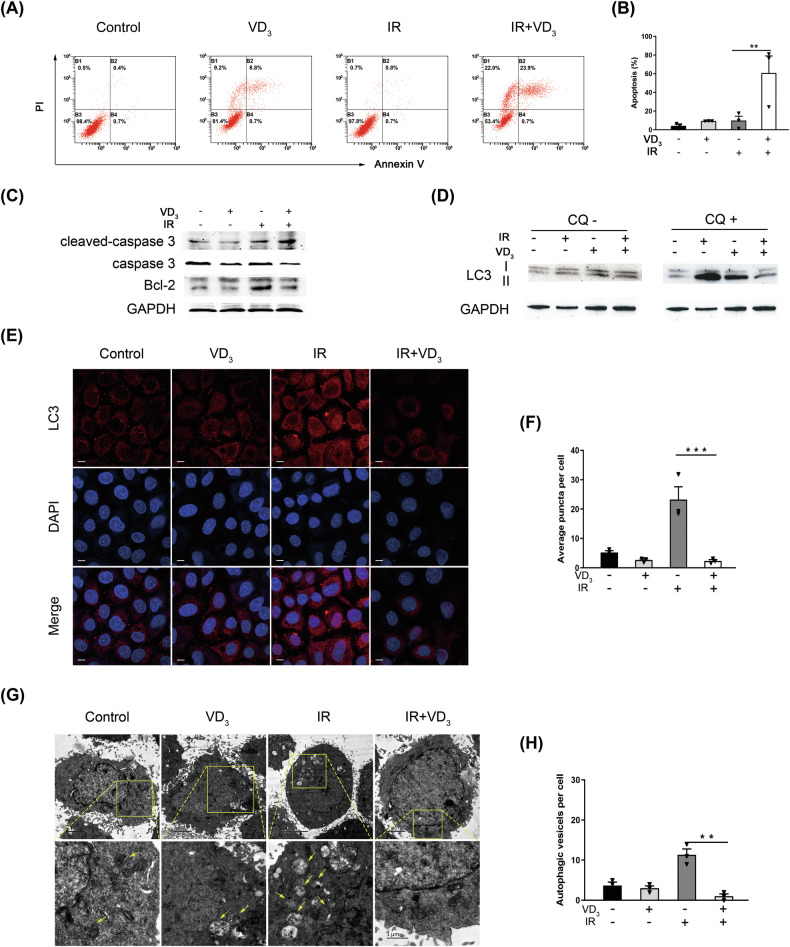
Vitamin D combining with radiation increased apoptosis and inhibited autophagy in cervical cancer. **A** Apoptosis analysis of Siha cell with indicated treatments. Cells were pretreated with vitamin D or radiation and then analyzed by Annexin V/PI. **B** Quantitative analysis of apoptotic rate. **C** The expression of apoptosis proteins was assessed by western blot. **D** Autophagy protein was determined by western blot. **E** Immunofluorescence of LC3 puncta with the indicated treatments. Scale bar: 20 μm. **F** Quantitative analysis of LC3 puncta. **G** Representative transmission electron microscope images of Siha cells under indicated conditions. Scale bar: 1 μm. Yellow arrowheads: autophagosomes. **H** Quantitative analysis of autophagosomes. *p < 0.05, **p < 0.01, ***p < 0.001, ****p < 0.0001.

In programmed cell death, apoptosis is a typical type of tumor cell death in response to therapeutics [13]. By detecting flow cytometry, we found that vitamin D or radiation alone had no significant influence on apoptosis, but the apoptotic proportion increased remarkably with the combination treatment (Fig. 5A, B). Further protein detection displayed that pro-apoptotic protein cleaved caspase 3 was upregulated in the combined treatment group, accompanied with a reduction in total caspase 3 and Bcl-2 (Fig. 5C). The above results were consistent with the proteome data and indicated that vitamin D could increase the apoptosis of CC cells in response to radiation.

As an effective balance system in cells to relieve stress, autophagy can be induced by conventional therapeutics [14]. We firstly examined the LC3 expression and found that the conversion of LC3I to LC3II was enhanced by radiation but declined seriously when combined with vitamin D, and this reduction of autophagy flux was exhibited more obviously with the addition of CQ (Fig. 5D). Furthermore, the formation of intracellular LC3 puncta appeared to be the same trend (Fig. 5E, F). It seemed that vitamin D could induce slight autophagy but radiation greatly boosted the autophagy, however, there was almost no autophagy flux after combining the two. In this context, there might be an antagonistic effect of combination treatment on autophagy. Accordingly, the formation of autophagosomes was further observed under the transmission electron microscope. Parallel to the results of immunofluorescence and protein, there was almost no autophagosome when performed with combined treatment in spite that vitamin D or radiation alone could induce autophagy (Fig. 5G, H).

Autophagy routinely maintains intracellular homeostasis by degrading intracellular harmful substances, and the impact of autophagy on tumors is distinguished under diverse external stress [15]. Autophagy was induced in CC when tumor cells were implemented with either single treatment but was counteracted under the combined treatment, which seemed contradictory. Nonetheless, the role of autophagy played in tumors is not designated, and a single form of cell death cannot decide the destiny of the tumor cell. Additionally, radiotherapy combined with vitamin D might potentially alter the form of autophagy, shifting cells from radiotherapy-induced protective autophagy to cytostatic autophagy. The detailed mechanism still requires further exploration. The above results presented that vitamin D combining with radiation ultimately increased apoptosis and inhibited autophagy.

### Vitamin D-induced radiosensitivity was regulated through the apoptosis mediated by Ambra1-dependent autophagy

**Fig. 6:**
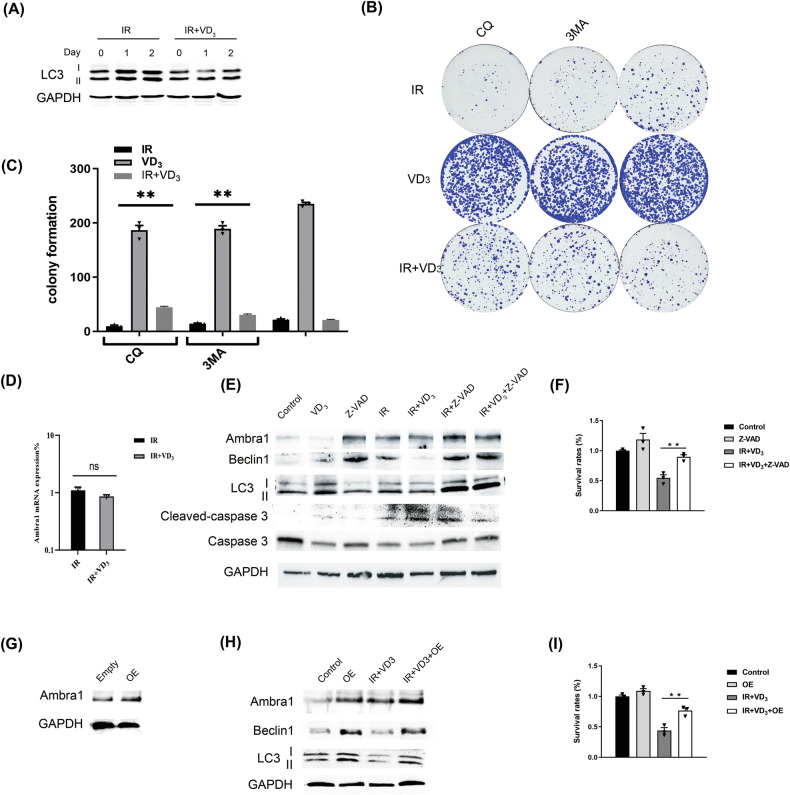
Vitamin D-induced radiosensitivity was regulated through the apoptosis mediated by Ambra1-dependent autophagy. **A** The expression of LC3 was assessed by western blot in a time-dependent manner. **B** Colony formation of Siha cells treated with indicated treatments and colonies were counted after incubating for 14 days. **C** Quantitative analysis of colonies. **D** Quantitative PCR of AMBRA1 mRNA comparing radiation and combination-treated Siha cells. **E** Western blotting of autophagy and apoptosis proteins in Siha cells dealt with combination treatments in the presence or absence of Z-VAD-FMK and the cell survival analysis (**F**). **G** Lentiviral infected Siha cells (OE) were tested by western blot. **H** Siha cells infected with lentivirus for indicated treatments were tested for autophagy protein by western blot and the analysis of cell survival (**I**). *p < 0.05, **p < 0.01, ***p < 0.001, ****p < 0.0001.

Firstly, constant monitoring of LC3 levels exhibited that radiation could induce autophagy, while vitamin D reduced this autophagy in a time-dependent manner (Fig. 6A). Based on this, we aim to explore which type of autophagy is induced by radiotherapy and the occurrence of autophagy in cells during radiotherapy combined with vitamin D. Accordingly, We assessed the proliferation of irradiated cells treated with autophagy inhibitors BafA1 and CQ. It was worth noting that these inhibitors blocked autophagy flux rather than prevented autophagy initiation [16]. The results ascertained that the ability of tumor suppression in the combined group was significantly reversed by the inhibitors, suggesting that radiation-induced autophagy played a cytoprotective role in our study (Fig. 6B, C). As a result, the initial proliferation inhibition effect of combination treatment was conversely relieved and showed the opposite tendency. This may be due to vitamin D converting radiation-induced protective autophagy into cytostatic autophagy, while autophagy inhibitors suppress this type of autophagy.

In order to further reveal how autophagy was downregulated by vitamin D, we firstly utilized qPCR to analyze the mRNA level of Ambra1 under different treatments. Intriguingly, the results displayed that slight alteration in transcription level made no significance and was unparallel to protein expression (Fig. 6D), which declared that the down-regulation of Ambra1 by combination of vitamin D and radiation might be derived from the protein post-translational modification. According to previous studies, the protein regulation of Ambra1 can be mediated by caspases cleavage or calpain-dependent degradation [17]. Then, we used apoptosis inhibitor Z-VAD to examine whether the stability of Ambra1 was affected by the apoptosis process. Surprisingly, we found that the downregulation of Ambra1 in the combined treatment group was significantly rescued when apoptosis was inhibited (cleaved caspase 3 significantly decreased), which was equally occurred in autophagy-related proteins Beclin-1 and LC3 (Fig. 6E). Driven by the above consequences, we tested whether this Ambra1-dependent autophagy regulated the vitamin D-induced radiosensitivity in CC. The results demonstrated that Z-VAD significantly reversed the survival inhibition of tumor cells in the combined group (Fig. 6F), which indicated that inhibition of tumor cells induced by the combination of vitamin D and radiation was significantly abrogated once apoptosis was inhibited.

**Fig. 7:**
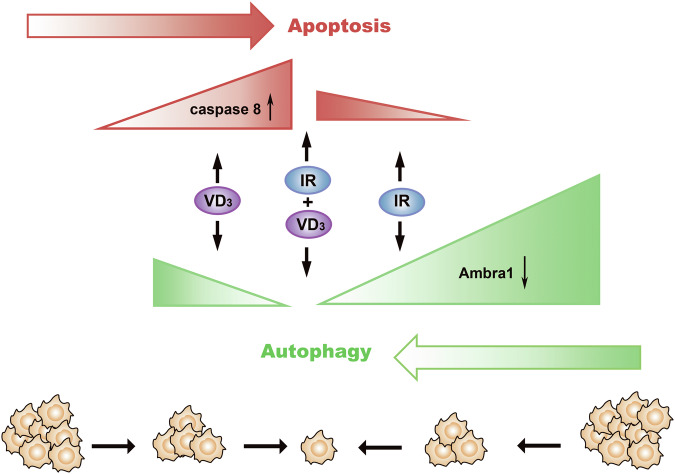
Schematic illustration of vitamin D-induced radiosensitivity in CC. Through the upregulation of apoptosis (caspase 8), vitamin D (VD_3_) reduced the cytoprotective autophagy (Ambra1) induced by radiation (IR) and enhanced the radiosensitivity in CC.

To further verify whether Ambra1-dependent autophagy or apoptosis performed a more influential role in the above results, we employed a lentiviral vector expressing Ambra1 (OE), and the expression of Ambra1 protein was confirmed (Fig. 6G). As expected, the OE upregulated the expression of LC3 and Beclin-1 (Fig. 6H) and significantly counteracted the inhibitory effect on tumor cell survival (Fig. 6I). More importantly, this reversal was similar to the utilization of Z-VAD. The consolidation of which caspase is responsive to the regulation of Ambra1 will be further explored in the future. All these results suggested that with the application of vitamin D, radiation-induced cytoprotective autophagy was inhibited and accompanied with the upregulation of apoptosis, and the ultimate significant enhancement in radiosensitivity was predominantly mediated by autophagy-related protein Ambra1 (Fig. 7). The Ambra1 expression was also validated in tumor tissues (Fig. S4).

## Discussion

With more and more epidemiological and preclinical studies paying attention to the role of vitamin D in cancer prevention [18, 19], people have begun to investigate the mechanism of antitumor effects in different cancers. In our study, CC patients in VDR-high had a worse prognosis compared to those in VDR-low, which suggested a strong correlation between VDR and CC. As the classic ligand of VDR, vitamin D was found to share many common targets with CC by the network pharmacology, which indicated that vitamin D could react more actively in VDR-high CC. Next, we revealed that a magnified therapeutic effect could be produced when radiation was combined with vitamin D, which was currently known as the first time to verify that vitamin D could sensitize CC to radiation both in vitro and in vivo.

The cell death caused by radiation is generally pointed to DNA double-strand damage and G2/M phase arrest [20]. However, our study has found that vitamin D did not cause the killing of CC cells through these two classic ways. In recent years, proteomic sequencing has gradually become a crucial technical method to investigate molecular mechanisms. Our proteomic results showed that apoptosis elevation and autophagy reduction occurred simultaneously after the combination treatment, indicating that these two phenotypes were closely linked to the radiosensitization of vitamin D. Furthermore, the apoptosis-related protein caspase 8 increased significantly along with the obvious decrease of autophagy-related protein Ambra1, suggesting that there was an interaction between these two phenotypes. As a traditional programmed cell death, apoptosis has been known for a long period and which is commonly mediated by the activation of cysteine protease (caspase) [21]. Caspases usually appear as inactive precursors, which can cleave the target protein once activated [22]. In our study, vitamin D or radiation alone did not cause substantial apoptosis, but a combination of both induced apparent apoptosis. This result revealed that combining vitamin D and radiation could deliver a synergistic effect by initiating a significant apoptosis effect in CC.

Autophagy, as a stress response to external pressure, its impact on tumor cells is not appointed; it may be cytotoxic, cytoprotective, or non-protective [23]. Each form of autophagy has its own characteristic. Therefore, by using autophagy inhibitors, we found that radiation-induced autophagy was cytoprotective in CC, and the role of vitamin D was to relieve this protective autophagy. Likewise, when we utilized apoptosis inhibitors to block the activation of caspase family, we found that Ambra1 expression was significantly restored, implying that caspases were involved in the downregulation of Ambra1. At the same time, cell viability inhibited by the combined treatment was also rescued, indicating that caspase affected vitamin D-mediated radiosensitization. Furthermore, the synergistic tumor suppression of combination treatment also exhibited the same trend with caspase inactivation when performed with Ambra1 overexpression. All these premises deduced the intricate regulation between caspase and Ambra1.

The cell death in response to therapeutics cannot be classified discretely, and different forms usually co-occur in tumor cells when receiving external pressure. Ambra1 can bind with Beclin1 to stabilize the Beclin1/Vps34 complex and promote autophagosome formation [24]. Some studies have reported that the COOH terminal fragment of Beclin1 can be transferred to mitochondria, causing the release of Cytochrome C and promoting the positive feedback of cell apoptosis [25]. Similarly, Ambra1 was also reported to be an essential target for caspase to inhibit autophagy [26]. Therefore, we speculate that the initiation of apoptosis triggered by vitamin D can lead to site-specific cleavage of autophagy proteins. That is, activated caspase 8 inhibits the formation of the Beclin1/VPS34 complex by cleaving Ambra1; simultaneously, the cleaved fragments of autophagy-related protein can bind to the mitochondrial membrane to propagate apoptosis. However, this proposal still requires to be further verified by subsequent experiments.

Our previous studies discovered that vitamin D could intensify the radiation efficacy by regulating EMT in colorectal cancer [7], which may be attributed to the metastatic expansion characteristics of colorectal cancer cells. Nonetheless, our current study revealed that autophagy occupied a more critical position in CC. A study reported that E6 and E7 proteins of HPV could act on autophagy by the modulation of Ambra1 [27]. We suppose that Ambra1 might be modulated by HPV to trigger an antagonistic or synergistic consequence for vitamin D-induced radiosensitization in CC. Nonetheless, what is the interactive contact between radiation and HPV? CC is one of the few tumors which can be prevented by antivirus and radiotherapy exhibits a crucial role in CC treatment. Some literature has reported that their association may derive from the regulation of human immunity [28]. Therefore, the potential anchor between HPV status and radiotherapy is worth deep exploration in CC. Ultimately, our study confirmed the considerable value of vitamin D in radiotherapy of CC and unveiled the underlying mechanism. It provided an empirical basis for using vitamin D as an adjuvant to improve the radiotherapeutic effect of CC and delivered adequate evidence for locating susceptible targets and clinical biomarkers in the future.

## Materials and methods

### Cell lines

Siha and Caski human CC cells were used. Siha cells were cultured in DMEM medium (RPMI-1640 for Caski) supplemented with 10% fetal bovine serum and 1% penicillin-streptomycin solution. For all cell culturing, standard conditions (5% CO_2_ and 37 °C) were used.

### Ambra1 stable overexpression cell lines

pCMV-EGFP-3FLAG lentiviral vector specific for human Ambra1 (OE) was purchased from OBiO, for Ambra1-overexpressed Siha cells, 8 µg/mL puromycin was added to the medium as the maintaining agent, and the expression efficiency was evaluated by western blotting with Ambra1 antibody.

### Cell treatments

Cells of appropriate amounts were seeded in 6-well or 96-well plates and the cultured CC cells were pretreated with ethanol (Control), vitamin D (VD_3_, 100 nM), radiation (IR, 6 Gy), and combination treatment (IR+VD_3_). To evaluate autophagy, cells were treated with 25μM chloroquine (CQ) or 100 nM bafilomycin A1 (BafA1) for the indicated time as recommended. Caspases activity was inhibited by Z-VAD-FMK (Z-VAD) at the concentration of 20 μM for 12 h.

### Antibodies

The primary antibodies against GAPDH, cleaved-caspase3, caspase 3, γH2AX, Beclin-1, caspase 8, and cleaved-caspase8 were from Cell Signaling Technology. Antibodies against Bcl-2, and VDR were from Santa Cruz Biotechnology, and LC3B was from Sigma. HRP-conjugated secondary antibodies used were purchased from Cell Signaling Technology: anti-rabbit (5151), anti-mouse (5257).

### IR exposure

After pre-incubation with vitamin D, cells were exposed to the X-ray radiation involving the 6-MeV X-ray photon at 200 cGy/min produced by a linear accelerator (Elekta, Sweden). The distribution of radiation was corrected by a 1.5-cm-thick bolus.

### Radiosensitivity assay

Colony-formation assay was used to detect the growth inhibitory effects of vitamin D and radiation on cells in six-well plates. Appropriate cells were seeded into culture plates and then dealt with indicated treatments for 10–14 days. Finally, colonies were fixed with methanol and stained with 1% crystal violet. Colonies containing more than 50 cells were recorded. The surviving cell fraction was calculated and analyzed as previously described [7].

### CCK-8 proliferation assay

An amount of 2 × 10^3^ cells/well were seeded in 96-well plates and the viability of treated cells was assessed by CCK8 assay (Beyotime, China). The amount of the living cells was examined by measuring the absorbance at 450 nm with BioTak Elx808.

### Flow cytometry

Apoptosis staining was performed using Annexin V-FITC kit (Sungenebiotech, China), and the cell cycle was stained with propidium iodide (Sigma, USA). Stained cells were analyzed using Becton cytometer (Becton CYTOMINCS FC500, USA). We recorded the percentage of both early and late apoptotic cells as the cell death and analyzed the DNA content with Multicycle AV DNA software.

### Immunofluorescence analysis

Treated cells were fixed with 4% paraformaldehyde for 20 min at room temperature, permeabilized with 0.5% Triton X-100 in PBS for 10 min, and then blocked with 5% BSA in PBS for 30 min. After washings, cells were incubated with anti-γH2AX or anti-LC3B primary antibodies (all 1:100) at 4 °C overnight, then incubated with anti-rabbit TRITC-conjugated secondary antibody (Thermo Fischer) for 1 h at 37 °C. Before the measurement, samples were stained with DAPI (Abcam, UK) for 5 min. Immunofluorescences were examined with Olympus FV1000 confocal microscopy. The number of γH2AX or LC3B localizing dots was analyzed by using GraphPad Prism.

### Western blotting analysis

Harvested cells of a six-well plate were lysed with radioimmunoprecipitation (Beyotime, China) containing 1× protease inhibitor cocktail. Proteins collected were centrifuged at 14000 rpm at 4 °C for 10 min and quantified using Protein Assay Dye Reagent Concentrate (Bio-Rad, USA). The total proteins were separated by electrophoresis performed on SDS-PAGE gel and transferred to a nitrocellulose membrane (Amersham, Germany). The membranes were blocked with 5% skim milk for 1 h at room temperature or bovine serum albumin and then probed with the specific primary antibodies under agitation at 4 °C overnight. After washings, the membranes were incubated with appropriate secondary antibodies for 1 h at room temperature. For the analysis, all resolved protein bands were detected using the Odyssey system (LI-COR Odyssey, USA).

### Quantitative proteomics

Label-free quantitative proteomics was used to quantify the dynamic changes of the treated samples as previously described [7]. Siha cells (1 × 10^7^) were treated with indicated conditions and then processed to the LC-MS to construct the bioinformatic analysis. The cutoff of the fold-change for differentially expressed proteins was set >1.5 or <1/1.5 and the *p*-value < 0.05 was deemed significant.

### Transmission electron microscopy

The harvested cell samples were processed as described previously [CRC paper] by the Public Technology Platform of Basic Medicine, Jilin University School of Medicine. The sections acquired by LEICA EM UC7 ultramicrotome were observed under an FEI TECNAI SPIRIT electron microscope.

### Real-time PCR

RNA was extracted using Trizol (Invitrogen) and an RT-kit (Promega) was used for cDNA synthesis. qPCR was performed with the SYBR Green qPCR Mix (Biosharp).

Primers: human AMBRA1 forward (5′-CTCTTCCTCAGACAACCAGGGT-3′) and reverse (5′-TCCAAGCGAAGGTGCAGACATC-3′), GAPDH level was used as an internal control, forward (5′-GTCTCCTCTGACTTCAACAGCG-3′) and reverse (5′-ACCACCCTGTTGCTGTAGCCAA-3′).

### Xenograft mouse model

To create the Siha xenograft mouse model, Balb/c female nude mice (4–6 weeks old) were subcutaneously injected into the right hind limb with 1 × 10^7^ Siha cells. While the volume of the tumors reached 50 mm^3^, the mice were treated with 2 μg/kg of vitamin D intraperitoneally or PBS, respectively, once every 2 days for a total of 2 weeks. The radiation treatment consisted of a 10 Gy of electron beam when the volume reached 100 mm^3^. Tumor size was measured every 2 days and the measurements were recorded as the largest superficial diameter (A) and the smallest superficial diameter (B). Tumor volume was calculated using the following formula: *V* = *A*^2 ^× *B*/2. Paraffin-embedded tumors and organs were H&E stained. Histopathology examinations were accomplished using a NIKON ECLIPSE E100 microscope.

### Statistical analysis

Data are shown as means ± standard deviations (SDs) of at least three independent experiments. *P* < 0.05 was considered significant. Statistical analysis of immunofluorescence, PCR, and FACs data was undertaken using unpaired, two-tailed Student’s *t*-test. All statistical analyses were performed using GraphPad Prism 8 software.

## Supplementary information

Original Data

supplementary

## Data Availability

All data generated or analysed during this study are included in this published article and its supplementary information files.
