# Supplementary material for: Vitamin D sensitizes cervical cancer to radiation-induced apoptosis by inhibiting autophagy through degradation of Ambra1
Source: Cell Death Discov. 2025 Jan 4;11:1. doi: 10.1038/s41420-024-02279-7 (PMC11698873; doi:10.1038/s41420-024-02279-7)
Supplement: Supplementary file 2 — supplementary [file 41420_2024_2279_MOESM2_ESM.doc]

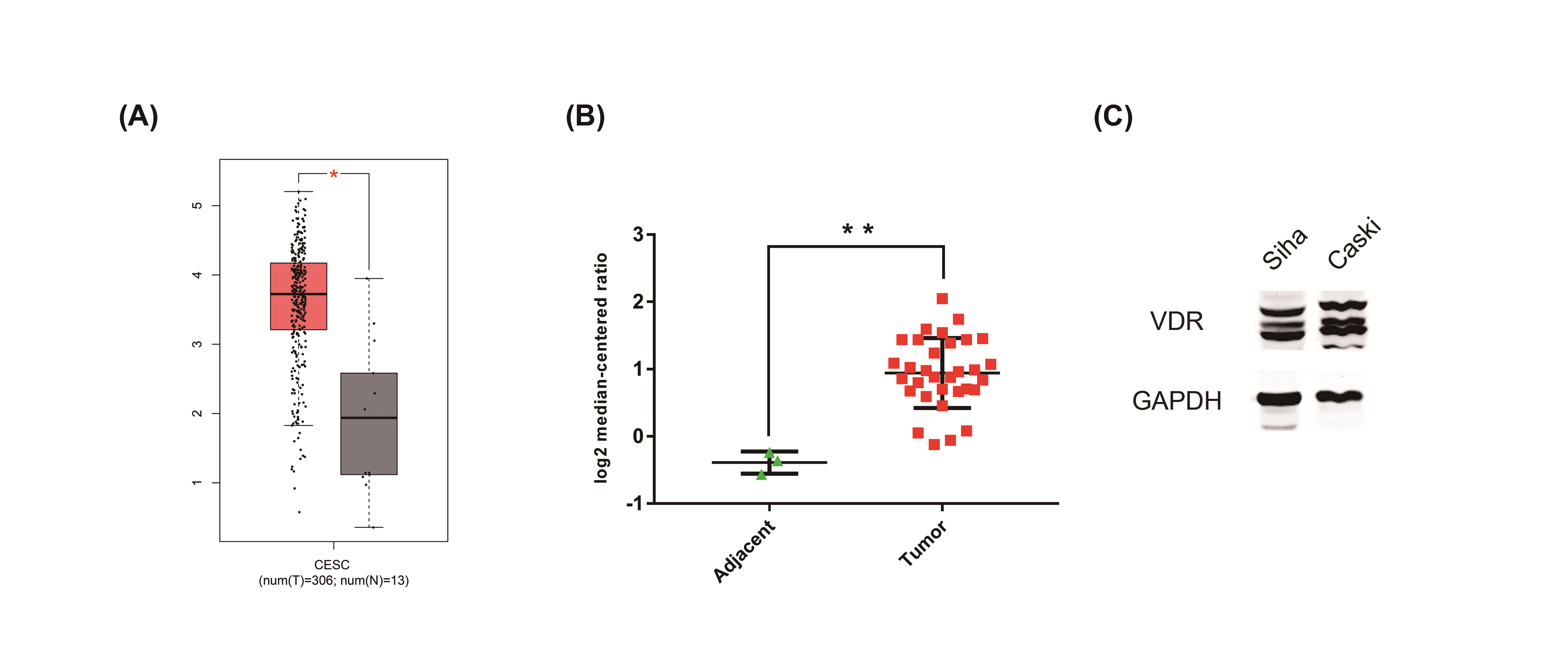


**Figure S1 VDR correlated with cervical cancer**

(A) Tumoral expression of VDR in the GEPIA database of 306 CC tumors and 13 adjacent tissues revealed significantly greater levels of VDR expression in CC tumors. (B) Representative samples of VDR expression in a cohort of Oncomine database. (C) Western blotting analysis of VDR proteins detecting in CC cells.


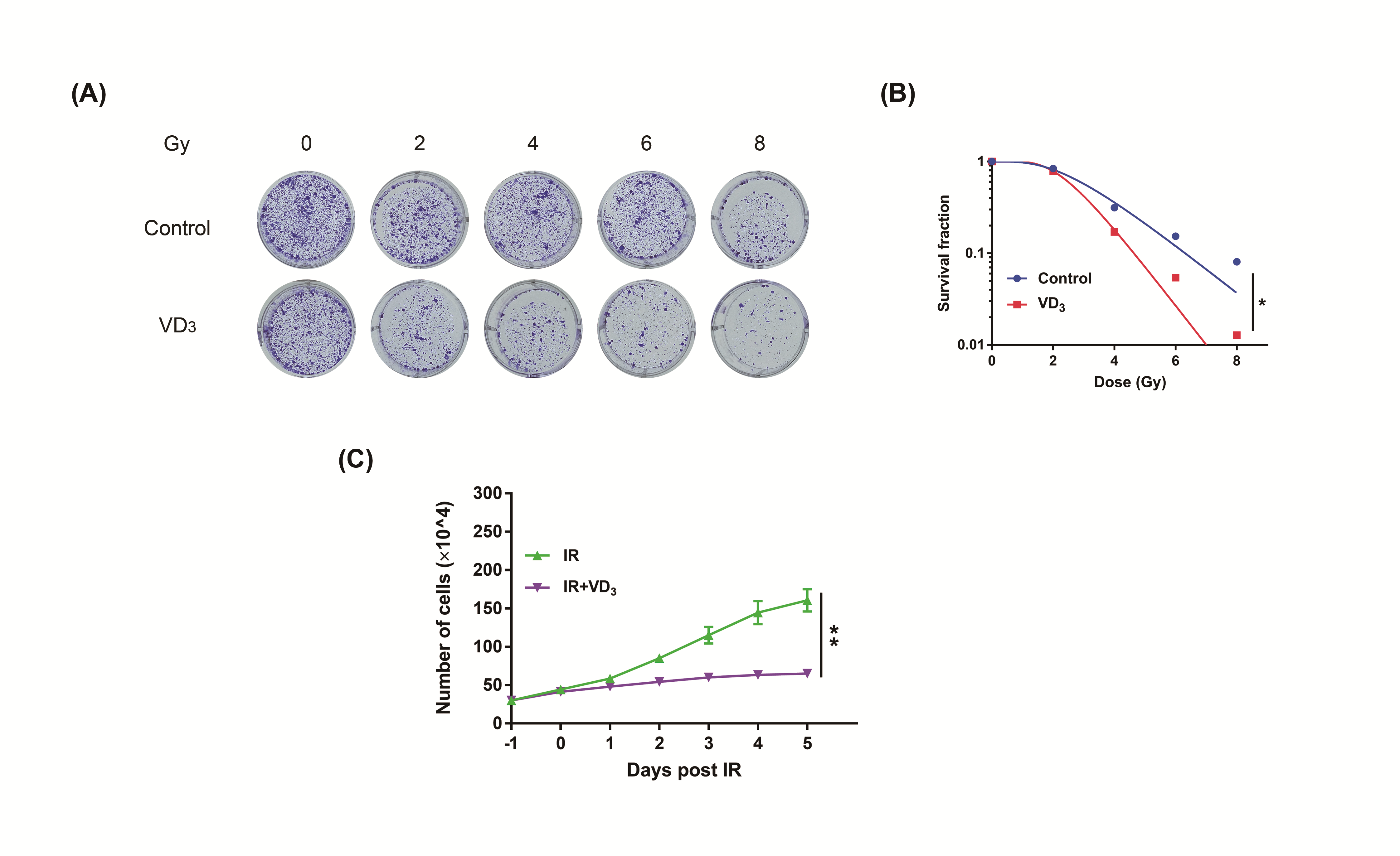


**Figure S2 Vitamin D enhanced the radiosensitivity of cervical cancer**

(A) A certain number of Caski cells were seeded in 6-well plates and then treated with radiation 0, 2, 4, 6, and 8 Gy or combined with vitamin D; 2 weeks later, crystal violet staining was utilized to calculate the number of clones. (B) The survival score curve of the obtained clone number. (C) Caski cells were planted in a 6-well plate and dealt with respective treatments. The number of surviving cells was measured by trypan blue staining every day for 5 days totally.


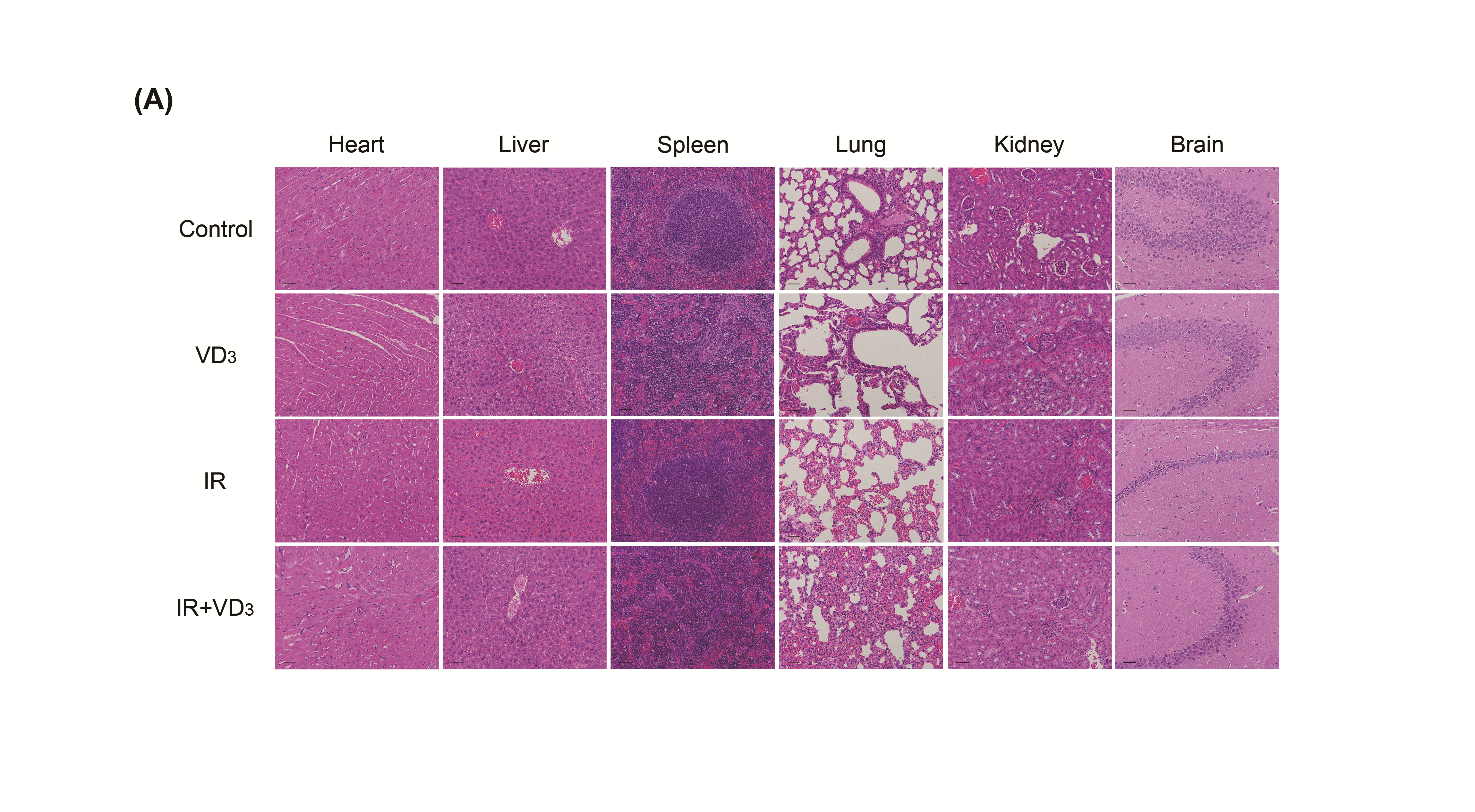


**Figure S3 Vitamin D enhanced the radiosensitivity of cervical cancer without additional toxic effects**

(A) H&E staining of the heart, liver, spleen, lung, kidney, and brain. Scale bar: 20 μm.


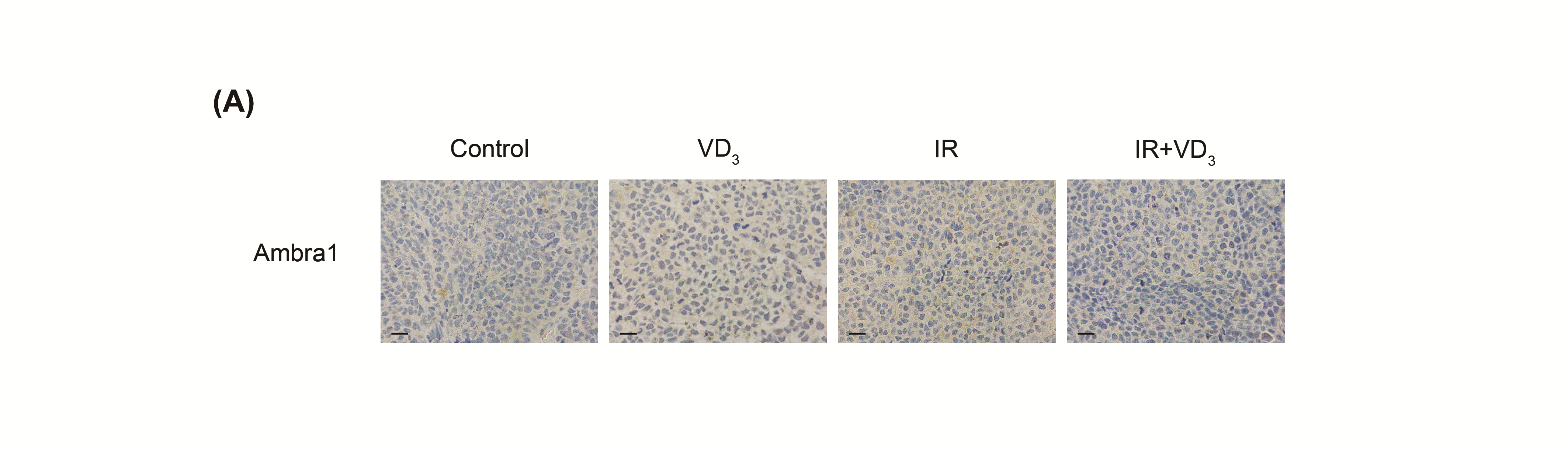


**Figure S4 Vitamin D combined with radiation downregulated Ambra1 in vivo.**

(A) Immunohistochemical (IHC) staining of Ambra1 in xenograft tumors. Scale bar: 20 μm.
