# Supplementary material for: Vitamin D sensitizes cervical cancer to radiation-induced apoptosis by inhibiting autophagy through degradation of Ambra1
Source: Cell Death Discov. 2025 Jan 4;11:1. doi: 10.1038/s41420-024-02279-7 (PMC11698873; doi:10.1038/s41420-024-02279-7)
Supplement: Supplementary file 1 — Original Data [file 41420_2024_2279_MOESM1_ESM.docx]

Dear Editor,

Regarding the Western blot images in our manuscript, some of the images were partially cropped in the preliminary experiments. We have now revised this and uploaded the original, uncropped images from our later experiments to the original data file. Additionally, we would like to clarify that some uncropped Western blot images from previous experiments are not available. However, we have made every effort to include the complete images for the vast majority of the Western blots. Thank you for your understanding.

Best regards,

Guanghui Cheng

**Figure 4**

Picture E

**Figure 5**

Picture D

**Figure 6**

Picture E

Ambra1

Beclin1

LC3

Cleaved-caspase3

caspase3
